# Lessons from the evaluation of the UK's NHS R&D Implementation Methods Programme

**DOI:** 10.1186/1748-5908-2-7

**Published:** 2007-02-19

**Authors:** Bryony Soper, Stephen R Hanney

**Affiliations:** 1 Health Economics Research Group, Brunel University, Uxbridge, Middlesex, UK

## Abstract

**Background:**

Concern about the effective use of research was a major factor behind the creation of the NHS R&D Programme in 1991. In 1994, an advisory group was established to identify research priorities in research implementation. The Implementation Methods Programme (IMP) flowed from this, and its commissioning group funded 36 projects. In 2000 responsibility for the programme passed to the National Co-ordinating Centre for NHS Service Delivery and Organisation R&D, which asked the Health Economics Research Group (HERG), Brunel University, to conduct an evaluation in 2002. By then most projects had been completed. This evaluation was intended to cover: the quality of outputs, lessons to be learnt about the communication strategy and the commissioning process, and the benefits from the projects.

**Methods:**

We adopted a wide range of quantitative and qualitative methods. They included: documentary analysis, interviews with key actors, questionnaires to the funded lead researchers, questionnaires to potential users, and desk analysis.

**Results:**

Quantitative assessment of outputs and dissemination revealed that the IMP funded useful research projects, some of which had considerable impact against the various categories in the HERG payback model, such as publications, further research, research training, impact on health policy, and clinical practice.

Qualitative findings from interviews with advisory and commissioning group members indicated that when the IMP was established, implementation research was a relatively unexplored field. This was reflected in the understanding brought to their roles by members of the advisory and commissioning groups, in the way priorities for research were chosen and developed, and in how the research projects were commissioned. The ideological and methodological debates associated with these decisions have continued among those working in this field. The need for an effective communication strategy for the programme as a whole was particularly important. However, such a strategy was never developed, making it difficult to establish the general influence of the IMP as a programme.

**Conclusion:**

Our findings about the impact of the work funded, and the difficulties faced by those developing the IMP, have implications for the development of strategic programmes of research in general, as well as for the development of more effective research in this field.

## Background

To achieve optimal care for their patients, healthcare systems must actively promote the quick transfer of sound research evidence into practice. None do so consistently and comprehensively [1-3]. The question of how to achieve effective research implementation is a key feature of the World Health Organisation's analysis of health research systems [4], and recent studies reinforce the desirability of looking at research implementation in relation to specific health care systems [5].

In the UK, the gap between research and practice remains wide despite a considerable, and rapidly growing, literature on research implementation [6]. What is wrong? The answer risks sounding trite. Implementing research findings is hugely complex, and we still have too little grasp of that complexity. This paper examines the recent history of research implementation in the UK through the lens of an evaluation of the National Health Service Research and Development (NHS R&D) Implementation Methods Programme (IMP) [7].

### History

The NHS R&D IMP was developed in 1994, and was an early attempt to explore in depth the issues of research implementation [8]. It was also the last in a series of time-limited, national NHS R&D programmes, and followed a well-developed model for setting priority topics and commissioning research. This model had been developed to address research needs in fields such as cardiovascular disease and mental health, and was largely clinically-focused, with an emphasis on randomised control trials (RCTs) as the gold standard research methodology. In contrast, the IMP covered a new, different, and very complex field which had not previously been systematically explored, spanning a wide range of behavioural, social science, management, science policy, and health service interests. There was a need to think beyond the clinical model.

Two groups were established to develop and support the IMP: an advisory group and a commissioning group.

The IMP advisory group advised the Central Research and Development Committee (CRDC) of the NHS on priorities for R&D in implementation research. It was established in October 1994, and disbanded 6 months later once that task had been completed. Members were drawn from the relevant academic community, and also included senior NHS staff and users representatives. The advisory group obtained information from:

- consultations with the NHS, its users, and the research community, and with the non-health sector.

- research overviews (health and non-health).

- reports from four specially convened working groups on the role of consumers, the media, changing clinical practice, the impact of policy and financial levers.

- expert papers, commissioned from outside experts and advisory group members.

The advisory group set 20 priority areas (Table 1) that were subsequently ratified by the CRDC, and developed research briefs describing each area and outlining the research approaches thought to be needed [8].

**Table 1:** Implementation Methods Programme priority areas, number of applications and projects funded in each area

**Ist Commissioning Round**	
**IMP 1 Influence of source and presentation of evidence on its uptake by health care professionals and others **(20 applications, 1 funded)	
IMP 1–11 Study of GP reasons for changing/not changing prescribing behaviour	Qualitative
	
**IMP 2 The principal sources of information on health care effectiveness used by clinicians **(11 applications, 1 funded)	
IMP 2–11 Nurses' use of research evidence in decision-making	Qualitative
	
**IMP 3 The management of uncertainty and communication of risk by clinicians **(21 applications, 4 funded)	
IMP 3–5 Communicating risk reduction to patients and clinicians in the secondary prevention of ischaemic heart disease	Qualitative & RCT
IMP 3–10 Investigation of Doctors' ability to understand and use clinical prognostic models when different metrics are used to describe model performance	Qualitative
IMP 3–12 Self-medication and the communication of risk: The case of deregulated medicines	Qualitative
IMP 3–16 Systematic review of risk communication – improving effective clinical practice and research in primary care	SR
	
**IMP 4 Roles for health service users in implementing research **(26 applications, 3 funded)	
IMP 4–13 Availability of information material to promote evidence-based patient choice	Qualitative
IMP 4–16 Evaluating improvements in the ability of health information services to provide information on clinical effectiveness	Qualitative
IMP 4–21 Effective communication – an evaluation of touchscreen displays providing information on prenatal diagnosis	RCT
	
**IMP 5 Why some clinicians but not others change their practice in response to research findings **(45 applications, 3 funded)	
IMP 5–23 Understanding the reasons for change, or not, in clinical practice – the case for dilatation and curettage	Qualitative
IMP 5–40 Uptake of effective practices in maternity units	Qualitative
IMP 5–41 Social networks and the use of research in clinical practice	Qualitative
	
**IMP 6 The role of commissioning in securing change in clinical practice **(16 applications, 0 funded)	
	
**IMP 7 Professional, managerial, organisational and commercial factors associated with securing change in clinical practice, with a particular focus on trusts and primary care providers **(35 applications, 0 funded)	
	
**IMP 8 Interventions directed at clinical and medical directors and directors of nursing in trusts to promote evidence-based care **(9 applications, 0 funded)	
	
**IMP 9 Local research implementation and development projects (such as GRiPP) **(17 applications, 0 funded)	
	
**IMP 10 Effectiveness and cost-effectiveness of audit and feedback to promote implementation of research findings **(16 applications, 2 funded)	
IMP 10–11 Evidence based secondary prevention of heart disease in primary care: an RCT of three methods of implementation	RCT
IMP 10–16 Effectiveness and costs of guidelines, prioritised audit criteria, and feedback in implementing change	RCT
	
**IMP 11 Educational strategies for continuing professional development to promote the implementation of research findings **(34 applications, 3 funded)	
IMP 11–10 Effectiveness of education & implementation strategies and the adoption of evidence based developments in primary care	RCT
IMP 11–26 Using informal learning in the implementation of research findings	Qualitative
IMP 11–29 Effectiveness of contin. education conferences and workshops to improve practice of health professionals	SR
	
**IMP 12 Effectiveness and cost-effectiveness of teaching critical appraisal skills to clinicians, patients/users, purchasers and providers to promote uptake of research findings **(18 applications, 2 funded)	
IMP 12-8 Systematic review of studies of effectiveness of teaching critical appraisal	SR
IMP 12-9 RCT of the effectiveness of critical appraisal skill workshops on health service decision makers in SW region	RCT
	
**IMP 13 The role of undergraduate (pre-qualification) training in promoting the uptake of research findings **(19 applications, 0 funded)	
	
**IMP 14 The impact of clinical practice guidelines in disciplines other than medicine **(33 applications, 1 funded)	
IMP 14–32 Review of the effectiveness of guidelines in professions allied to medicine	SR
	
**IMP 15 Effectiveness and cost-effectiveness of reminder and decision support systems to implement research findings **(22 applications, 7 funded)	
IMP 15-4 RCT of a simple prompting system re appropriate management of iron deficiency anaemia and its influence on clinical outcome	RCT
IMP 15-8 Effectiveness of computerised advice on drug dosage in improving prescribing practice	SR
IMP 15-9 Evaluation of computerised guidelines for the management of two chronic conditions	RCT
IMP 15-11 Cochrane SR of effects of paper & computer-based reminders and decision support on clinical practices & patient outcomes	SR
IMP 15-12 Comparing patient held prompt & reminder card to a doctor held prompt & reminder card to improve epilepsy care in the community	RCT
IMP 15–19 Maternity Guidelines Implemented on Computer (MaGIC)	Pilot study
IMP 15–21 Review of economic studies of reminders and decision support systems	SR
	
**IMP 16 The role of the media in promoting uptake of research findings **(20 applications, 2 funded)	
IMP 16–18 Systematic review of the impact of mass media campaigns on health services utilisation and health care outcomes.	SR
IMP 16–19 The role of the media in public and professional understandings of breast cancer	Qualitative
	
**IMP 17 Impact of professional and managerial change agents (including educational outreach visits and local opinion leaders) in implementing research findings **(16 applications, 2 funded)	
IMP 17-12 Is the involvement of opinion leaders in the implementation of research findings a feasible strategy?	Qualitative
IMP 17-13 Prevention of deep vein thrombosis: A feasibility study for a randomised trial of three different strategies to implement evidence based guidelines	Pilot study
	
**IMP 18 Effect on evidence – based practice of general health policy measures **(3 applications, 0 funded)	
	
**IMP 19 The impact of national guidance to promote clinical effectiveness **(16 applications, 1 funded)	
IMP 19-15 The injecting drug taker and the community pharmacist: impact of new department of health guidelines and obstacles to a broader service-providing base	Qualitative
	
**IMP 20 Analysis of use of research-based evidence by policy makers **(7 applications, 0 funded)	
	
**Joint DoH funded project (under IMP 4)**	
Evaluation of informed choice leaflets in maternity care	RCT
	
**2nd Commissioning Round**	
**Evaluation of the effectiveness of interventions to improve the uptake of research findings in practice**	
IMP R2-25 Evaluation of effectiveness and cost-effectiveness of audit, feedback and educational outreach in improving nursing practice and health care outcomes	RCT
IMP R2-34 Development and preliminary evaluation of decision interventions decision	Pilot study
IMP R2-64 RCT of dissemination & implementation strategies for guidelines for extraction of third molar teeth	RCT

The IMP commissioning group was established in 1995 to advise on the scientific merit and value to the NHS of the applications submitted, on the progress of commissioned work, and on the quality and value to the NHS of the IMP as it developed. There was some overlap in membership between the two groups. Two rounds of commissioning were undertaken, the first in 1995, the second in 1997. In total, 35 projects were funded fully by the IMP, and one project, on informed choice leaflets, was joint-funded (with the Department of Health). Thirty-two of the projects were based in universities, two in NHS Trusts, and two in charities. As was the usual practice, the IMP was at this point managed by an NHS regional office.

The IMP was always intended to be time-limited. Expectations initially were that it would reflect the timescales and budgets of existing programmes (e.g., a five-six year programme with a budget of £8 million). But by 1996, and in the context of the Culyer review [9], the strategic approach of the NHS R&D Programme as a whole was being reconsidered. A cap of £5 million was put on all time-limited national NHS R&D programmes, and funds for the second round of IMP commissioning were curtailed. In the same year, the IMP commissioning group was disbanded. As a result, the second round of commissioning in 1997 was undertaken by a subgroup of the original commissioning group, and this second round addressed just one priority area identified from gaps in the profile of the programme (Table 1) [10].

In 2000, responsibility for the IMP passed to the National Coordinating Centre for the NHS Service Delivery and Organisation R&D Programme (NCCSDO). In 2002, NCCSDO funded the Health Economics Research Group (HERG), Brunel University, to undertake a brief evaluation of the IMP (funded to £20K). The aim was to explore the quality of the outputs of the programme and the commissioning process, and to determine what lessons could be learnt for future commissioning and communication strategies. A full report of the evaluation is available [7].

## Methods

A range of quantitative and qualitative methods was used and triangulation techniques were applied.

### Documentary analysis

Analysis of published IMP documents was supplemented by a review of IMP files, exploring the development and work of the IMP, and the statements applicants made initially about the potential users of their research.

### Questionnaire to the thirty-six lead applicants of IMP-funded projects

A questionnaire was sent to all 36 lead applicants of IMP-funded projects. There was an extensive process to encourage participation, including a second posting to non-respondents followed up by selective emails and phone calls where necessary. The questionnaire was based on one developed previously by HERG for the evaluation of health R&D projects and programmes [11,12]. Questions covered knowledge production, each project's contribution to research training and further research, and the possible impact of research findings on health policy and practice. In relation to the questions on whether any research training and further research resulted from participation in an IMP project, an attempt was also made to assess the level of any contributions that came from the IMP project by inviting researchers, using their own opinions, to classify the contribution as considerable, moderate or small. Limited resources precluded any attempt to assess final outcomes in terms of benefits such as health gains, but the questionnaire did explore ways in which the dissemination of the research findings from IMP projects might have contributed to their impact, and the role of the IMP as a whole in this.

### Questionnaires to potential users on the dissemination and use of research findings

While the projects were funded on the basis of their contribution to the study of implementation methods, many of them were conducted in relation to specific medical fields and some projects shared a common theme. We were therefore able to group some of the projects by subject matter, and for each of four partially overlapping groups (women's health, the management of heart disease, shared decision making, and maternity care), a short questionnaire was designed. In each case the questionnaire supplied information about the three or four IMP-funded projects in the particular field, including the abstracts from the most important article from each project. Each questionnaire sought information from selected recipients about the dissemination and potential use of the research findings in that group. Three of the four questionnaires developed were distributed electronically (to 535 addresses for the three groups of recipients in total). The fourth survey, based on the three projects that related to maternity care, was posted to 207 heads of midwifery and 20 university researchers in perinatal care.

### Desk analysis

One aim of the study was to identify both the number and quality of the publications deriving from the programme. Previous analysis has demonstrated that it is not always sufficient to rely on the information about specific project publications returned by researchers [11]. Some additional review was therefore conducted of the articles that were claimed to have come from projects funded by the programme. Various databases were interrogated to assess aspects of the research outputs from the IMP. Citation analysis was undertaken for journal articles using the science and social science citation indices from Thompson's Institute for Scientific Information, and the relevant journal impact factors were recorded.

### Interviews

Twenty-five semi-structured interviews were conducted with members of the NHS R&D IMP advisory and commissioning groups. All but one of those approached for an interview agreed to participate, and in some cases they had served on both groups. The interviews focussed on the commissioning process and 15 of 20 commissioning group members participated. Of the remaining five, two had died, two were abroad and one could not be located. Limited resources meant that in total only 12 of 19 advisory group members were interviewed. In some instances those interviewed had successfully applied for funding from the IMP, and there was also discussion about the impact from their specific project. The interviews were recorded, transcribed, and entered into a database in which the coding frame was based on the semi-structured interview schedule.

## Results

### Quantitative assessment of outputs and dissemination

Data collection and analysis were informed by the HERG framework for assessing health research payback, and the various stages of that model are used here to present the quantitative data [13,14]. The final response rate to the questionnaire to lead applicants was 30 out of 36 (86%).

#### Publications

By Autumn 2002, there had been 120 publications that were a specific product of IMP funding. The numbers of the various types of publication are shown in Table 2. These figures are taken from two sources. First, from the 30 completed questionnaires and second, for the remaining six projects, from the publications listed in the earlier programme report of 2000 [10], provided they acknowledged IMP funding. Of the 59 articles in peer-reviewed journals, 41 were in journals given a journal impact factor by Thompson's Institute for Scientific Information. The journal used most frequently for publication, the BMJ, is also the one with the highest journal impact factor of those publishing articles from the programme. The journal most used that did not have an impact factor was The British Journal of Midwifery. While the recent publication dates of many articles reduced the value of citation analysis, some of the publications from projects that completed early had been widely cited according to the Science Citation Index. The article most cited, Coulter et. al., had been cited on 138 occasions (and over 200 times on Google Scholar) by Autumn 2006 [15]. Another much-cited publication arose from an early commission by the IMP advisory group to assist its discussions, and had, by Autumn 2006, been cited about 600 times on the Science Citation Index [16].

**Table 2:** Publications from the Implementation Methods Programme's 36 projects

**Type of publication**	**Number**
Peer reviewed journal article	59
Journal editorial	3
Journal letter	2
Published abstract	15
Book	2
Chapter	11
Non-peer reviewed article	2
Published conference proceedings	6
Publicly available full report	6
Other	14
**TOTAL**	**120**

#### Further research

Table 3 provides details (from the 30 completed questionnaires) of 15 follow-on projects funded from other sources, such as the Medical Research Council (MRC), but connected to IMP projects and undertaken by IMP researchers. In total such funding came to over £1.3 million. Case study two (described below) provides an important example of follow-on work that was clearly further research on implementation. Some non-IMP researchers have also built on the IMP projects.

**Table 3:** The importance of the Implementation Methods Programme research to securing funding for 15 further projects

	**Considerable importance**	**Moderate importance**	**Small importance**	**Contribution not recorded**	**Combined totals**
Number of projects where funding known	3	4	3		10
					
Total amount awarded in each category	£678K	£576K	£60K		£1,314K
					
Number of projects where amount of further grant not stated	2	2		1	5

Total number of projects	5	6	3	1	15

#### Research training

One of the difficulties facing the IMP was the lack of research capacity in this field. It is, therefore, particularly important to note that at least nine projects involved research training. An accepted indicator of such research training is whether it has led, or will lead, to higher/research degrees [13,17]. The degrees obtained by researchers associated with these nine projects include four PhDs and three MDs (Table 4) based on the 30 completed questionnaires. Table 4 also shows the level of contribution to the research degree that came from the IMP project.

**Table 4:** Qualifications gained or expected from involvement in a project funded by the Implementation Methods Programme

**Qualification**	**Obtained**	**Expected**	**Contribution of the IMP project to the qualification**		
			
			**Considerable**	**Moderate**	**Small**
MSc	1			1	
MPhil	1		1		
MD	3		2	1	
PhD		4	2	2	

#### Impact on research, teaching and clinical practice: views of potential users

The responses from the three electronic questionnaires to potential users of IMP research findings were too low (22 out of 535) to provide results that could be widely generalised. Of course, this low response rate could be interpreted as a lack of knowledge about the IMP as a whole. There was a better response to the postal survey sent to midwives. A summary of the figures from the 100 out of 227 questionnaires returned (44%) is shown in Table 5.

**Table 5:** Knowledge and use of the Implementation Methods Programme by midwives and by perinatal care researchers

	**Number**	**% of those returned**
Questionnaires distributed	227	
Questionnaires returned	100	
Knew about IMP	35	35%
Heard of at least one project	80	80%
Read an article from at least one project	68	68%
Findings from at least one project already influenced:		
a) clinical practice	54	54%
b) research	8	8%
c) teaching	20	20%
Findings from at least one project will influence:		
a) clinical practice	73	73%
b) research	14	14%
c) teaching	24	24%
Findings from at least one project have/will have influenced others	73	73%

The response rate is clearly not sufficiently high to provide figures that can be viewed as properly representative. Nevertheless, they do suggest a reasonably high level of knowledge about the projects. In particular, the project on informed choice leaflets [18] was known by more than half of the heads of midwifery who replied, and most had read at least one of the articles about it. A few respondents pointed out that our evaluation questionnaires themselves had provided a good means of disseminating information about these projects. Given the widespread knowledge about some of the projects, and some of the comments made, it seems reasonable to suggest there is quite a considerable interest within the midwifery profession in the implementation of research findings. This interest would also appear to follow through into practice. About half the respondents claimed that their clinical practice was already being influenced by the findings of some projects, and about two-thirds thought this would be so in future.

One of the problems when interpreting the level of existing and potential impact was the question of what exactly was being referred to when discussing impact in relation to projects from the IMP. It is possible that some replies to the questionnaire related to whether the midwives had been directly influenced by existing research on the substantive topic, rather than whether they had been influenced by the conclusions of the IMP project, which, at a meta level, had examined ways to encourage the implementation of this existing research. Nevertheless, 44 midwives, for example, thought that the findings from the project on the uptake of effective practices in maternity care [19] might in future be used in their unit to influence clinical practice. Other midwives, however, explained that the findings of the IMP studies would not impact on them because they already knew about the substantive research in question and were already implementing it, or they knew about the substantive research as well as the IMP findings, but lacked the resources required to implement these findings.

#### Impact on teaching, and on health policy and practice: views of IMP researchers

The project files revealed extensive claims made in project applications about potential users and about the benefits that would flow from the projects. The questionnaire responses received from lead researchers gave the current situation. There were more claims of possible future impact than of existing impact, by a factor of approximately 2:1. This is shown in Table 6. The results are broadly in line with other national NHS R&D programmes [20-22], and suggest that impact on health policy and practice from the majority of the projects has as yet been tentative. The two case studies described below provide examples of the type of impact on teaching/policy/practice produced directly as a result of the IMP project and the full report provides further examples [7].

**Table 6:** Lead researchers' opinions about the existing and potential impact of their Implementation Methods Programme project (n = 30)

**Type of impact**	**Number of projects making an impact**			
	**Yes**	**No**	**Don't know**	**Blank**

Already impacted on policy	9	15	2	4
Expect to impact on policy	16	11	0	3
Already impacted on practice	8	12	5	5
Expect to impact on practice	17	5	1	7

#### Dissemination

Collectively the dissemination of results from IMP projects was not systematically organised. However, there was activity by individual researchers at project level, including 92 presentations primarily to academic audiences and 104 presentations to practitioners and/or service users.

We undertook an analysis of presentations reported by early 2000 [10]. This gave the ratio of national to international presentations as 5:2.

### Qualitative findings from interviews with advisory and commissioning group members

The 25 interviews with members of the IMP advisory and commissioning groups focused on the development and commissioning of the research programme and on the overall influence of the IMP.

#### An innovative programme – understanding implementation

As a research topic, research implementation differs from more clinically orientated research, necessarily involving a wider range of disciplines and methodological approaches. Members of the IMP advisory and commissioning groups were aware of the challenge this posed, and intended to be wide-ranging and innovative. But many subsequently felt that they had underestimated the difficulties involved in developing a research agenda in a new and relatively complex field [7]. There were also considerable time pressures. As a result, and despite extensive interdisciplinary discussion in both groups, a "clinical tendency" remained within the IMP. This had an impact on priority setting and on the research finally commissioned, and prompted innovative attempts to develop RCTs to address the complex issues raised by the IMP, but also raised concerns about the lack of real engagement with the social sciences.

#### Setting and developing priority areas for research

There was considerable variation between the IMP priority areas. Interviewees said that in some areas it was clear what was needed and what research approaches were available, in others more exploratory work was needed, and in some areas it was too early to fund anything. But at the outset, it was not clear which area was which. As one interviewee put it:

"...it could be that, given our knowledge at the time, let's consult and find a whole series of areas, a scattergun approach, and some of the areas do seem sensible but others less so, and let's just see who comes up with good projects and go for it and try and get some flowers to bloom".

The first round of commissioning was, to this extent, biased to what was thought to be achievable. Studies were funded in 13 out of the 20 priority areas (see Table 1).

To inform subsequent calls for research, a member of the commissioning group undertook an overview of funded studies after the first round of commissioning [23]. This compared the priority areas with what had been offered and with what was subsequently funded, and found varying degrees of overlap between the 20 priority areas (also recognised by the advisory group [8]). The author identified considerable overlap between areas 1 and 2, with 2 being a subsection of 1, and noted that areas 6, 7 and 8 were "a 'super area' linked along the purchaser/provider axis". In his view this overlap had allowed too much leeway in the subject matter of applications, resulting in the commissioning group being unable to fund studies in certain areas. Other interviewees confirmed this view, talking about "a lack of clarity in what we wanted", the difficulties of knowing what to study in relation to, for example, health service commissioning within a changing political context, the lack of established networks between researchers and NHS managers, and complex methodological challenges. Few of these areas were amenable to straightforward clinical trials.

In the light of its findings, the overview made recommendations about future funding in each priority area. The subsequent curtailment of the programme meant that this analysis could not be used as intended.

#### Commissioning research – composition of the commissioning group

Interviewees praised the way the commissioning group had been encouraged to approach the complex tasks it faced. But they also drew attention to underlying difficulties. Time pressures were often seen as a limiting factor, although a minority of interviewees actually thought these had been beneficial, helping to focus minds. A more fundamental pressure was the dual requirement to assess both the quality of research applications *and *their relevance to the NHS. Those best able to undertake the first task (researchers) are not necessarily those best able to undertake the second (NHS commissioners and practitioners) and, on occasion, their views differed.

#### Commissioning research – interaction with applicants

The quality of many of the applications was poor. As a consequence, the commissioning group put a great deal of effort into developing applications, helping to build research teams, and provide methodological support. Two workshops were held with applicants, and commissioning group members spent considerable amounts of time brokering these arrangements [10].

There was general, but not complete, agreement about the need for this work. Those who questioned its value pointed to the (sometimes unfulfilled) expectations raised by repeated iterations between applicants and funding agencies, and to the difficulties research teams might experience working to briefs that had been developed for them by the commissioning group. Some interviewees thought it wrong to work with teams who had not already developed a strong methodological understanding; others thought that this was just what was needed to help researchers relatively new to a field. The commissioning group aimed to fund high-quality research, and was concerned about methodological rigour. Much thought went into the development of RCTs, and statistical advice was provided, as was advice on economic evaluation. This was regarded by those involved as useful and productive. The challenge was to establish a balance between funding only work of the highest quality and developing the research capacity. In the first round of commissioning, 28 projects were funded and four others that were asked to re-submit were eventually funded. No picture emerged of what happened to non-funded IMP applications, although we were told that "much of the stuff we turned down got funded by others".

Concerns were also raised about the timing of commissioning group/researcher interaction. Despite the acknowledged quality of the inputs, workshops for applicants tended to be awkward affairs, with competing teams reluctant to talk in each other's presence. But we were also told that exploratory workshops earlier in the process might not have attracted the full range of relevant disciplines or all potential applicants.

#### Commissioning research – avoiding bias and conflicts of interest

The IMP was the first programme within the NHS R&D Programme to look at change and management. Members of the advisory and commissioning groups agreed that a wide range of research approaches was needed in this field, but there were differing views about what this meant. Some thought that people with skills in the social sciences should be available to support those doing trials on guidelines; others saw a need to draw on existing bodies of knowledge in various social science disciplines and integrate them with NHS issues. Some thought it important to develop qualitative methodologies; others identified a raft of issues about the development of RCTs that led to considerable methodological gains, as well as attempts to stretch this approach (too far, some thought) to cover the many complex questions raised by the IMP. The IMP did not resolve these issues, but it did raise them and so helped to promote what has subsequently become a fruitful debate.

Many of the research projects funded by the IMP were systematic reviews, RCTs, or pilot studies for the latter, accounting for approximately two-thirds of the total expenditure [24]. Interviewees agreed, however, that this did not reflect undue bias. Reasons given for the tendency to fund this type of work included:

- the poor quality of many of the qualitative applications.

- concerns about generalisability.

- a related "failure to embrace complexity", a tendency to go for known and more mechanistic approaches, and not to pursue complex questions in unfamiliar territory.

- the need for research teams working in this field to have good links with the NHS, and existing clinical trial teams already had these links.

- the fact that, as one interviewee put it, "medics tend to favour RCTs".

#### Probity

Given the need to involve researchers in research commissioning and the limited pool of people available in some fields, it is not uncommon for those commissioning work to submit applications to their own programme. In this case, the success rate from members' institutions (9%) was comparable with the overall success rate (8%). Proposals involving members of the commissioning group as named applicants had a higher success rate, 55% (n = 12). Concerns about this, both *ante *and *post facto*, were raised by members with the commissioning body. The response was robust: this does not concern us as long as due process is followed. The NHS R&D Programme operations manual provided guidance and the commissioning group considered the question of probity prior to commissioning projects. Due process was followed and recorded.

#### Need for a communication strategy

The need to identify and involve the potential users of IMP research was clear from the outset. In this field, it was seen as particularly important. An advisory group briefing paper put the position clearly: 'As the advisory group is concerned with implementation presumably it should set something of an exemplar role in the active communication of its own work' [7]. But in the end, the dissemination of results from IMP projects was not systematically organised by the IMP as a programme. We were told that members of the advisory and commissioning groups were initially too overwhelmed with the immediate tasks of getting the programme up and running to give a communication strategy much attention. And then the commissioning group was disbanded early, and was not available to develop a coordinated approach.

### Examples of important studies

Drawing on the findings from the questionnaire survey to lead researchers, augmented by comments made in interviews, a more detailed account of two studies is provided here to illustrate the type of project funded, and the key findings and outputs. They were selected on the basis that they formed interesting studies illustrating important points and were projects for which the questionnaire respondent was also interviewed during the course of the project evaluation because of their role on the advisory and commissioning groups.

#### Case study 1: Availability of information to provide evidence-based patient choice [IMP 4–13]

This was a four stage study the aims of which were: to investigate the availability of patient information materials about treatment choices for ten conditions for which high quality systematic reviews existed; to assess the materials in terms of scientific validity and acceptability to patients; to develop guidance on the production of patient information; and to provide practical help to health authorities and health care providers on evidence-based patient choice [15]. The study found that current materials omitted relevant information, failed to give a balanced view of the acceptability of different treatments, and ignored uncertainties; many adopted a patronizing tone. It concluded that groups producing information materials must start with the needs defined by patients, give treatment information based on rigorous systematic reviews, and involve multidisciplinary teams (including patients) in developing and testing materials.

Thus, although the study found much at fault with current practice, it also produced clear and positive messages about possible improvements and translated these into practical advice for health authorities and health care providers. These positive, practice-orientated findings had considerable impact. They were actively disseminated by the research team through a series of meetings with potential users, and were subsequently used, for example by the British Heart Foundation to revise their leaflets. They were also presented in a book, *Informing Patients *[25], which was at one time the King's Fund's best-selling title: sales figures of over 1,300 are seen as excellent for a book in this category. And, as already noted, the main paper from this research was the most highly cited paper from an IMP-funded project.

#### Case study 2: Nurses' use of research evidence in decision-making [IMP 2–11]

This descriptive and analytic study used qualitative interviews, observation, and statistical modeling to explore the factors that influence nurses' access to, interactions with, and use of, research material in their decision-making processes in three large acute hospitals [26]. The main finding of the study was that nurses have the potential to participate in evidence-based decision-making, but that the presentation and management of research knowledge in the workplace poses significant challenges. A considerable educational, research, management and policy response is required if this potential is to be exploited. Specific recommendations covered: training nurses to handle uncertainty rather than to expect certainty; developing evidence-based change agents; organizing and increasing access to the knowledge needed for clinical decision-making. There has been a series of publications from the project, and it has influenced various courses and educational programmes. In addition, it helped to open up the previously under-explored field of research implementation within nursing and provide significant opportunities for further work, including a £339,000 MRC-funded study for the team to build on their original IMP project. It was entitled: *Nurses' use of research information in clinical decision-making in primary care*.

## Discussion

This evaluation was designed to explore the quality of the outputs of the IMP and of the commissioning process. Our findings regarding the impact of the work funded and about the difficulties faced by those developing that programme have implications for the development of more effective research in this field, both in the UK and elsewhere.

### Outputs of the programme

As is demonstrated by the examples just given, the IMP funded various useful research projects, some of which had considerable impact against the various factors in the HERG payback model, such as publications, further research, research training, impact on health policy and clinical practice.

### Developing and commissioning a research programme – lessons learnt

#### Setting and developing priority areas for research

The advisory group followed the pattern set in previous NHS R&D programmes and set 20 priority areas. In the end, studies were funded in only thirteen of these areas. The reasons given for this shortfall nicely summarise the state of implementation research at that time. Interviewees acknowledged a lack of clarity in what was required, and in part attributed this to the difficulties of knowing what NHS staff wanted. The converse was also true. When proposals did come from managerial groups or from clinicians previously unused to doing research, they often sought answers to policy-driven questions – important questions but hard to turn into research, especially in a rapidly changing political context. The picture is one of lack of engagement between researchers and NHS staff, and lack of understanding of each others' perspectives. There were few well-established networks through which researchers (particularly social science researchers) could access key NHS advisors, such as medical directors or directors of nursing. This made it difficult to mobilise people rapidly into large, rigorously designed studies. And even with all its collective expertise in this wide-ranging field, the commissioning group lacked direct knowledge of strong methodological groups in some priority areas.

We conclude that, in this particular field at this particular time, a programme of 20 priority areas was too ambitious. It would have been better to build the programme more gradually, addressing fewer priority areas in each round of commissioning. In our view, complex new research fields such as this would benefit from more preparatory work, lower initial expectations (especially as regards the pace of the programme) and the ability to re-visit and learn from early results.

#### The role of the commissioning group

When commissioning R&D for the NHS, the dual requirement to assess the quality of research applications and to consider their relevance to the NHS can lead to tensions. We suggest that commissioning groups should adopt protocols at their first meeting to cover the role and remit of members, taking account of members' differing backgrounds, skills, and experience, as well as defining the group's relations with external advisors.

#### Support for research applicants

It may be necessary to provide support for research applicants during the commissioning process in research fields that are relatively new and/or where the existing research capacity is weak. This was the approach adopted by the IMP and it has also, at different times and in various ways, been adopted by other funding agencies, including the MRC, the Economic and Social Research Council, and the NHS Health Technology Assessment (HTA) Programme. This support can be:

- to develop a precise, easily understood research brief prior to commissioning. As was attempted in the IMP, an iterative process to commissioning may also be required, building on detailed analysis of previous rounds of commissioning.

- to support the development of applications during commissioning. This was vigorously pursued in the IMP and thought by some interviewees to have been helpful, although views differed about the form and timing of this interaction.

- to assist project monitoring after commissioning. In the IMP, attempts were made to provide ongoing support for project development in relation to some funded studies. But, in the absence of an ongoing overall programme, these attempts were project-specific; no overall picture of these approaches and their impact has emerged.

Despite their general support, we found very divergent views among interviewees about the form this interaction with applicants should take. As one of them suggested, "this is something we need research on."

#### Communications

A tailor-made communications strategy was important for the IMP, as the advisory group recognised at the start, but one was never set up. The main barriers were time constraints and the subsequent history of the IMP. At programme level, we were told that the IMP did raise awareness of the need to improve the dissemination of research findings (in general) in the NHS. But most IMP researchers did not feel that being supported by the IMP, rather than funded as separate, isolated projects, had had any effect on the impact of their findings. In contrast, individual projects in other national R&D programmes gained credibility and attention from being part of a wider programme [21,22].

Yet the need for dialogue with users was, and is, clear. The advisory group recognised that their work was just a beginning:

*"further work will be needed to take forward this research agenda, with close dialogue between researchers, research funders and those working in and using services..." *and that: *"This interaction is also essential to encourage ownership of the results that emerge – to ensure implementation of the research agenda on implementation." *[8].

The key lesson from the IMP is that adequate communication is not an option; it is integral to implementation research. Future research programmes should be supported by effective communications strategies.

### Influence of the programme

Can the IMP, as one of our referees suggested, be itself considered an implementation of an organisational change? In one sense it can. The IMP was part of a highly innovative attempt, through the new NHS R&D Programme, to reshape the way in which R&D was prioritised, commissioned, and used by the service. But in a more important sense such a description makes too bold a claim for the IMP. Twelve years ago relatively little was understood about research implementation and why it sometimes worked well but mostly did not. The IMP was, first and foremost, an R&D programme charged with producing high-quality studies of value to the NHS in an under-explored field. It was this remit at this time that made the IMP so important, some would have said essential, within the NHS R&D Programme as a whole. The IMP was intended to contribute substantially to the development of the knowledge base from which the service could implement sound research effectively and so promote improvement. This is why it generated such enthusiasm among members of the advisory and commissioning groups.

The timing was crucial. Even if the IMP had received its full funding and run its full course with the continued involvement of the commissioning group as planned, the state of knowledge at that time would have limited its overall impact. This is no reflection on the quality or utility of the individual projects funded. But most research on implementation at that time focussed on single interventions aimed at the lone practitioner, and the IMP was no exception. It was only later, and after much more study, that the real complexities became clearer, and the need to look at research implementation in the round – across organisations and within local contexts – became obvious [1].

To this extent, the issues raised in advisory and commissioning group discussions concerning the scope and nature of implementation research, the appropriateness of different research methodologies, and the need for effective interaction between disparate disciplines, have influenced the debate, especially in the UK, ever since. By openly exploring those topics, and involving so many in that process, the IMP raised the profile of implementation research and increased interest and understanding. Twelve years after the IMP was established there is now real dialogue between academic clinicians and the social sciences, and wider appreciation of the need to use theoretical frameworks from disciplines such as cognitive psychology [27] and organizational studies [28,29] in implementation research. There is greater understanding of the complexity of research implementation and of the multi-faceted approaches required to achieve beneficial, sustainable improvements in clinical practice [30].

There are of course still difficulties. Publication of research findings on implementation and innovation in peer-reviewed journals, at least until very recently, could be problematic [31,32]; meta-analysis in this methodologically complex field is extremely challenging [33]; healthcare professionals are often reluctant to accept evidence that has not been derived from a randomised controlled trial [34].

One special difficulty is the organisational context within which implementation research operates. There is an irony here. The IMP was established at a time of large changes within the NHS and the NHS R&D Programme [7], and was itself shaped in unintended ways by the organisational changes around it. As more than one interviewee pointed out to us, continual re-assessment of the organisation of the NHS and consequent change is a major impediment to the successful implementation of research in the NHS. In this respect nothing has altered. If anything the scale and pace of change has accelerated – continual and rapid re-organisation has become endemic in the NHS.

What has altered is our understanding of how continual reorganisation impacts on the service, and on its ability to use good evidence from research. There is recognition of the price to pay in any reorganisation, especially in reorganisation that is not itself based on sound evidence [35]. Nevertheless, some changes, such as the establishment of bodies such as the National Institute for Health and Clinical Excellence (linked to the NHS HTA Programme) and the new NHS Institute for Innovation and Improvement, can be beneficial. NHS "buy-in" to the need for a sound evidence-base on research implementation *per se *has improved.

## Conclusion

The IMP was an early attempt to develop a systematic programme of implementation research for the NHS. The way in which the IMP was set up and developed was influenced by the various contexts in which it had to operate – the relatively new NHS R&D Programme, the developing internal market in the NHS, general health policies at the time, and so on. Some individual IMP projects have had considerable impact in their particular fields. The influence of the programme as a whole is less easy to assess although it is clear that it prompted important debate about the nature and scope of implementation research that has continued ever since.

This evaluation has highlighted important lessons about how research priorities should be set and work commissioned in a relatively new field, and, crucially, about how essential it is that research programmes such as the IMP are adequately supported by effective communication strategies.

If it were established today the IMP would be different. But should it be established today? Can such large-scale strategic funding approaches advance implementation science? There is no doubt about the importance of funding sound generalisable studies as the basis for sustainable improvement in the implementation of research, nor about the value of ongoing dialogue between researchers and users about the content and conduct of such work. Insofar as a strategic approach promotes such activities, we believe it is desirable. But any strategic approach should itself be based on what can be learnt from programmes such as the IMP.

## Competing interests

The authors declare they have no financial competing interests, but SH was a member of a team that made an unsuccessful bid for funding under the IMP.

## Authors' contributions

SH played a large role in devising the original evaluation and SH and BS were primarily responsible for conducting the evaluation. BS led on the production of this article and both authors read and approved the final manuscript.
